# A Novel FAPI-Based Radiopharmaceutical for SPECT Imaging of Fibrotic Interstitial Lung Disease

**DOI:** 10.3390/ph18121779

**Published:** 2025-11-23

**Authors:** Guangjie Yang, Jingnan Wang, Yu Liu, Jiyun Shi, Xueyang Zhang, Yangzhong Zhou, Qian Wang, Fan Wang, Li Huo

**Affiliations:** 1 Department of Nuclear Medicine, State Key Laboratory of Complex, Severe, and Rare Diseases, Center for Rare Diseases Research, Peking Union Medical College Hospital, Chinese Academy of Medical Science and Peking Union Medical College, Beijing 100730, China; yangguangjie@pumch.cn (G.Y.); jingnanwang1991@163.com (J.W.); liuyu@pumch.cn (Y.L.); 2 State Key Laboratory of Biomacromolecules, Institute of Biophysics, Chinese Academy of Sciences, Beijing 100101, China; shijiyun@med.uestc.edu.cn (J.S.); wangfan@bjmu.edu.cn (F.W.); 3 Department of Rheumatology and Clinical Immunology, State Key Laboratory of Complex, Severe, and Rare Diseases, Peking Union Medical College Hospital, Chinese Academy of Medical Sciences and Peking Union Medical College, Beijing 100730, China; zhxy97@outlook.com (X.Z.); hubertchow09@163.com (Y.Z.); 4 Medical Isotopes Research Center and Department of Radiation Medicine, School of Basic Medical Sciences, International Cancer Institute, Peking University, Beijing 100191, China

**Keywords:** fibroblast activation protein inhibitor (FAPI), single-photon emission computed tomography (SPECT), fibrotic interstitial lung disease (fILD), pulmonary fibrosis, molecular imaging

## Abstract

**Objectives**: Early and noninvasive detection of fibrotic interstitial lung disease (fILD) is a critical but unmet clinical necessity. This study aimed to evaluate the feasibility of using ^99m^Tc-HYNIC-Glu(PEG_4_-oncoFAPi)_2_ (denoted as ^99m^Tc-H-PoFP_2_), a novel ^99m^Tc-labeled radiopharmaceutical that targets fibroblast activation protein (FAP), for single-photon emission computed tomography (SPECT) imaging of pulmonary fibrosis in a mouse model and preliminary clinical studies. **Methods:** ^99m^Tc-H-PoFP_2_ could be conveniently afforded using a kit formula with high radiochemical purity and stability. The binding specificity and affinity of ^99m^Tc-H-PoFP_2_ for FAP were validated by an in vitro binding assay. The in vivo characteristics of ^99m^Tc-H-PoFP_2_ were also determined. **Results:** ^99m^Tc-H-PoFP_2_ was eliminated quickly via the urinary system, leading to low normal tissue uptake and a high target/background ratio. SPECT imaging demonstrated significantly enhanced uptake of the ^99m^Tc-H-PoFP_2_ in bleomycin-induced fibrotic lung tissues, with visual effects superior to those of normal mice. Thus, a pilot clinical study of ^99m^Tc-H-PoFP_2_ SPECT/CT imaging was conducted in 12 patients diagnosed with fILD. The physiological biodistribution of ^99m^Tc-H-PoFP_2_ in patients was predominantly observed in the kidneys, bladder, liver, and pancreas, with relatively minor accumulation in the thyroid, salivary glands, and spleen. fILD patients exhibited elevated pulmonary ^99m^Tc-H-PoFP_2_ uptake in the affected lung regions. Furthermore, the uptake of ^99m^Tc-HPoFP_2_ demonstrated moderate correlations with the results of pulmonary function tests (PFTs). A higher gender–age–physiology (GAP) index was associated with elevated standardized uptake value maximum (SUVmax) and target-to-background ratio (TBR) values. **Conclusions:** Collectively, this study demonstrates the potential of ^99m^Tc-HPoFP_2_ for SPECT imaging and assessing fILD by targeting FAP overexpressed in fibrotic lung tissues. This strategy offers new possibilities for noninvasive and precise assessment of pulmonary fibrosis.

## 1. Introduction

Fibrotic interstitial lung disease (fILD) is a chronic lung disease characterized by abnormal collagen accumulation in the lung parenchyma, leading to dyspnoea, progressive pulmonary dysfunction, and even death [1]. Once fibrosis is established, anti-fibrotic treatment merely delays the decline of lung function, leading to a median survival of 3–4 years [2]. Currently, clinical diagnosis of pulmonary fibrosis mainly involves pulmonary function tests (PFTs) and high-resolution computed tomography (HRCT) [3]. Although CT is capable of detecting morphologic changes in the diseased lung, its effectiveness is limited to relatively late stages of the disease when scar tissue formation is evident, and it cannot provide disease activity information in pulmonary fibrosis. Lung tissue biopsy and histopathological examination are the gold standards for diagnosis. However, this method is invasive and exhibits high sampling variability because of obtaining only a few lung tissues. In addition, these examinations cannot predict precisely the disease course in individual patients, which is often highly variable [4]. Therefore, there is an urgent need to develop a noninvasive method to monitor ongoing fibrosis and accurately identify progressors, which might significantly benefit the clinical management of fILD and their prognosis.

Nuclear medicine imaging can visualize biological processes at the molecular level in a noninvasive manner. Functional tissue imaging of fibrosis or fibrotic activity is able to complement the structural information afforded by HRCT. A promising marker for monitoring fibrogenesis is the fibroblast activation protein (FAP), a serine protease predominantly expressed on the membranes of activated fibroblasts under various pathological conditions, including cancer, fibrosis, inflammation, or wound healing. Its over-expression is confined to these pathologic sites and not typically found in most healthy tissue, making it an ideal target for radiotracers development [5]. Several fibroblast activation protein inhibitor (FAPI) based radiotracers have been developed for non-invasive diagnosis and quantitative evaluation of FAP expression in pulmonary fibrosis [6]. Preclinical and clinical studies using FAPI tracer imaging have yielded promising outcomes. Most of these radiotracers are labeled with either gallium-68 (^68^Ga) or fluorine-18 (^18^F) for positron emission tomography (PET) imaging. Despite the growing availability of PET imaging over the past decades, it remains inaccessible in some developing regions. In contrast, SPECT imaging offers a more cost-effective and convenient option with superior time-cost efficiency, possibly making it better suited for the current conditions in developing regions. Additionally, SPECT imaging involves a relatively lower radiation dose, which is advantageous for patients requiring serial follow-up examinations. The widespread use and longer half-life (6 h) of ^99m^Tc enable more flexible imaging windows than PET. However, limited investigations on ^99m^Tc-FAPI SPECT imaging in fILD were reported, with only one study conducted to date [7].

In this study, we developed a novel ^99m^Tc-labeled FAPI-based radiotracer, ^99m^Tc-HYNIC-Glu(PEG_4_-oncoFAPi)_2_ (denoted as ^99m^Tc-H-PoFP_2_), for SPECT imaging of pulmonary fibrosis. To obtain optimal diagnostic performance, we strategically introduced PEG4 linkers and constructed a homodimer structure. To our knowledge, the introduction of PEG4 linkers could improve the hydrophilicity and prolong the circulation time of radiopharmaceuticals [8], while the homodimer structure could strengthen the binding affinity through multivalent interactions with FAP overexpressed in fibrotic tissues [9]. Therefore, the designed structures were expected to obtain enhanced diagnostic effectiveness for lesion detection, with higher accumulation in the lesions and faster clearance of unbound tracers. Furthermore, we investigated the safety, biodistribution, and clinical feasibility of this optimized radiopharmaceutical in 12 patients diagnosed with fILD, with the aim of advancing noninvasive diagnostic methodologies for this debilitating condition.

## 2. Results

### 2.1. ^99m^Tc-Radiolabeling and In Vitro Evaluation of ^99m^Tc-H-PoFP_2_

**Figure 1:**
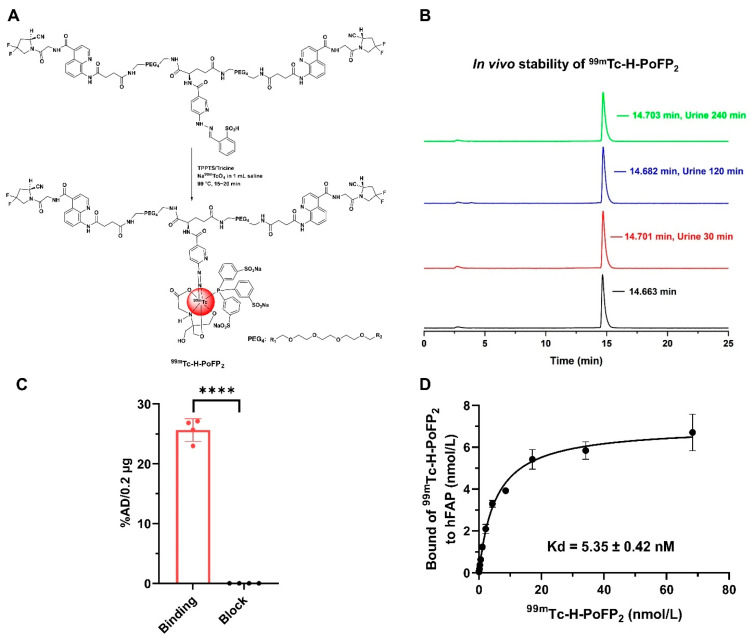
Radiolabeling and characteristics of ^99m^Tc-H-PoFP_2_. (**A**) H-PoFP_2_ was labeled with ^99m^Tc via a kit formula. (**B**) ^99m^Tc-H-PoFP_2_ was afforded without purification, and the in vivo stability of ^99m^Tc-H-PoFP_2_ was determined. The radiolabeling yield and radiochemical purity of ^99m^Tc-H-PoFP_2_ were determined by radio-HPLC. (**C**) The binding specificity to human FAP of ^99m^Tc-H-PoF_2_ was determined by protein binding assay. In the block study, ^99m^Tc-H-PoFP_2_ was incubated with unlabeled precursor H-PoFP_2_. **** indicates that *p* < 0.0001. (**D**) The binding affinity of ^99m^Tc-H-PoF_2_ to human FAP was determined by protein saturation binding assay. TPPTS: trisodium triphenylphosphine-3,3′,3″-trisulfonate.

The labeling precursor (H-PoFP_2_) and reference compounds were prepared successfully with high chemical purity (>95%) and identified with mass spectrometry (Figures S1 and S2). H-PoFP_2_ can be easily formulated as a kit for ^99m^Tc radiolabeling conveniently in routine clinical application, and the ^99m^Tc-labeling procedure could be completed within 25 min (Figure 1A). The in vivo stability of ^99m^Tc-H-PoFP_2_ was determined by testing the murine urine samples at different time intervals post-injection (p.i.). Negligible decomposition was observed, and the intact ^99m^Tc-H-PoFP_2_ was greater than 95% within the tested time (Figure 1B). ^99m^Tc-H-PoFP_2_ exhibited significant binding values (presented as percentage of the total added dose (%AD) per 0.2 μg hFAP with the number of 25.65 ± 1.88 for the binding group and 0.03 ± 0.01 for the block group, where *p* < 0.0001) (Figure 1C). The dissociation constant Kd of ^99m^Tc-H-PoFP_2_ to hFAP was 5.35 ± 0.42 nM (Figure 1D). Although the assay setup was different, the binding affinity of ^99m^Tc-H-PoFP_2_ was comparable to that of other FAPI-based radiotracers [9,10,11,12]. In addition, the partition coefficients (LogD) of ^99m^Tc-H-PoFP_2_ demonstrated high hydrophilic properties with the value of −3.56 ± 0.09, which aligned with the rapid clearance and short circulation half-life (T_1/2α_ = 3.02 min; T_1/2β_ = 166.10 min) (Figure S5).

### 2.2. ^99m^Tc-H-PoFP_2_ Showed Promising Detection Ability in Bleomycin-Induced Lung Fibrosis Model

**Figure 2:**
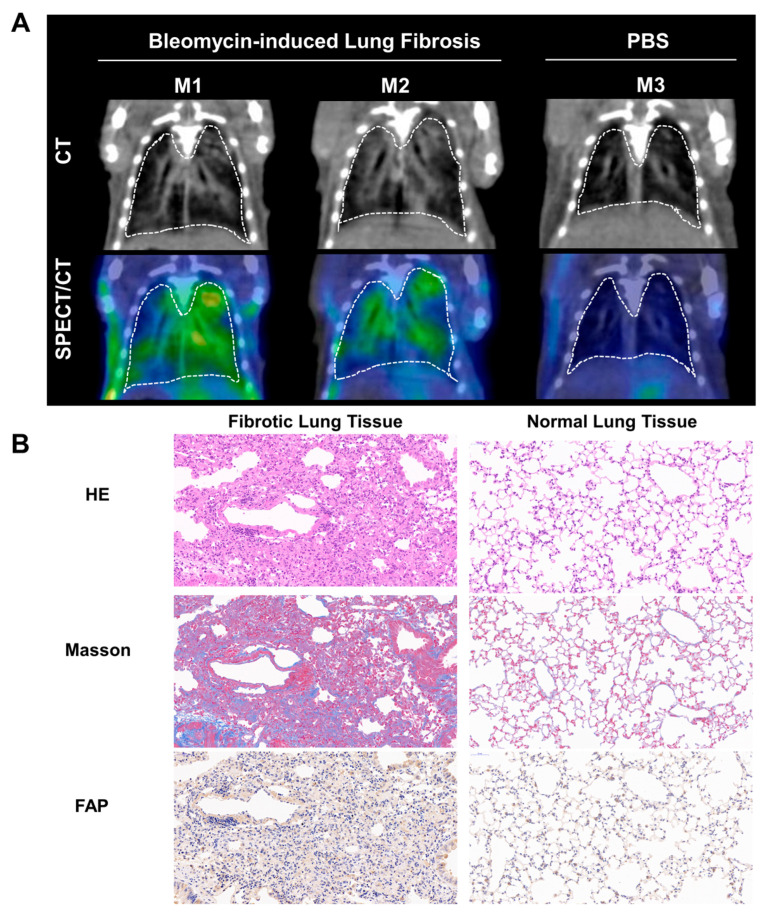
^99m^Tc-H-PoFP_2_ could detect fibrotic regions in bleomycin-induced lung fibrotic mice. (**A**) SPECT/CT imaging of ^99m^Tc-H-PoFP_2_ in bleomycin-induced lung fibrotic mice and normal mice (n = 3). (**B**) HE, Masson staining, and FAP immunostaining of murine fibrotic and normal lung tissue. (Left panel: 10×, right panel: 20×).

We explored the ability of ^99m^Tc-H-PoFP_2_ in the detection of the bleomycin-induced lung fibrosis model. In the mice with pulmonary fibrosis, obvious parenchymal regions in the lungs could be seen from the CT signals, which proved to be fibrotic lesions by hematoxylin and eosin staining (HE) and Masson staining (Figure 2A). Meanwhile, in the corresponding SPECT/CT images of these regions, accumulation of radioactive signals can also be visualized, suggesting that the regions where the probe accumulated were the pulmonary fibrosis regions. These regions also showed strong FAP expression by immunohistochemistry staining (IHC) (Figure 2B). On the contrary, in the CT and SPECT images of normal mice, no obvious fibrotic CT or radioactive signals could be seen. Additionally, whole-body planar SPECT/CT images showed the quick renal excretion and specific accumulation in fibrotic lung tissues with high target-background contrast (Figure S6). These results indicated that ^99m^Tc-H-PoFP_2_ SPECT/CT imaging had promising application values in the diagnosis of pulmonary fibrosis. Therefore, we also conducted a preliminary study in patients diagnosed with pulmonary fibrosis.

### 2.3. Dynamic Biodistribution in Organs and Dosimetry Analysis

**Figure 3:**
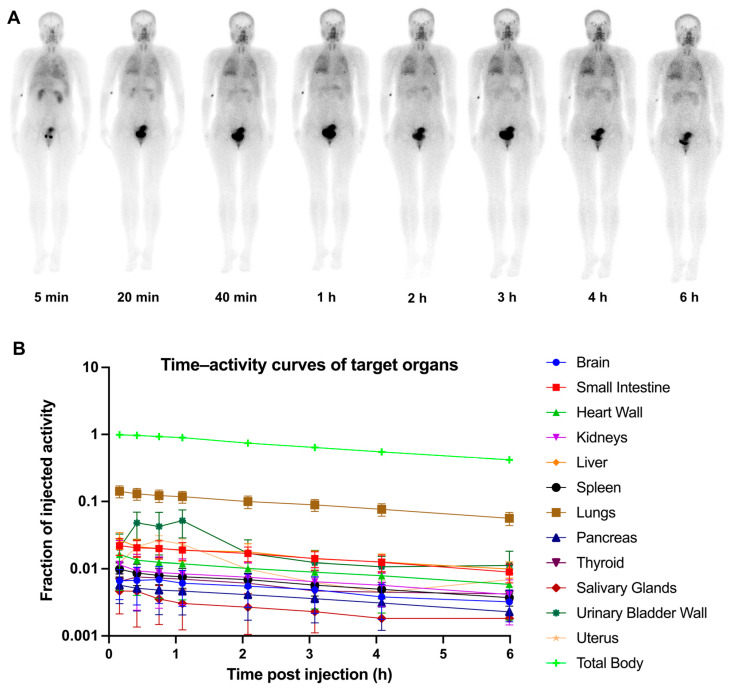
Serial whole-body anterior projection images (**A**) and time–activity curves of target organs (**B**) of a 34-year-old female patient at different time points (5 min, 20 min, 40 min, 1 h, 2 h, 3 h, 4 h, and 6 h p.i.) following intravenous administration of ^99m^Tc-H-PoFP_2_.

A summary of the demographic characteristics of 12 fILD patients is presented in Table 1. No adverse events associated with the administration of ^99m^Tc-H-PoFP_2_ (723.9 ± 52.4 MBq) were reported. ^99m^Tc-H-PoFP_2_ uptake was predominantly observed in the kidneys, urinary bladder, pancreas, thyroid, salivary glands, liver, and spleen. There was significant uptake in the chest regions of patients at the early time points, which remained visible even at 6 h p.i. A representative illustration of ^99m^Tc-H-PoFP_2_ distribution from whole-body scintigraphy was depicted in Figure 3A. Dosimetry calculation analysis was conducted in six representative patients using HERMES Hybrid Viewer 4.0 Dosimetry according to OLINDA/EXM (version 2.0) methodology in the guideline [13]. The regions of interest and geometric mean count of target organs were derived from sequential background-corrected anterior and posterior images. Time–activity curves of target organs were shown in Figure 3B. As shown in Table 2, organ-specific absorbed doses and effective doses for ^99m^Tc-H-PoFP_2_ were calculated with the OLINDA/EAM software (version 2.0), and the International Commission on Radiological Protection (ICPR) 103 tissue weighting factors were used for effective dose estimation. The average total-body effective dose was determined to be 0.00724 mSv/MBq.

### 2.4. Uptake of ^99m^Tc-H-PoFP_2_ in fILD Patients and Relationship with PFT and GAP Index

**Figure 4:**
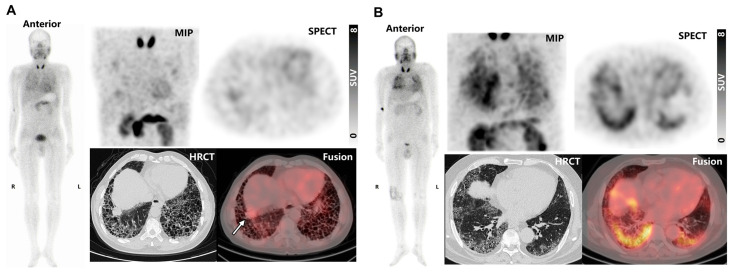
(**A**) ^99m^Tc-H-PoFP_2_ SPECT/CT and HRCT images of a 65-year-old male patient. The solid lesion in the right basal part was noted by a white arrow. (**B**) ^99m^Tc-H-PoFP_2_ SPECT/CT and HRCT images of a 63-year-old male patient. MIP, maximum intensity projection.

In fILD patients, SPECT/CT images revealed elevated ^99m^Tc-H-PoFP_2_ uptake predominantly in subpleural and peripheral areas, corresponding to pathologic regions of pulmonary fibrosis, including reticular abnormalities, traction bronchiectasis, and honeycombing as seen on HRCT. Exemplary cases are illustrated in Figure 4. In one instance (Figure 4A), massive honeycombing opacities were noted in the subpleural and peripheral regions, particularly in the basal parts, while mild elevation of ^99m^Tc-H-PoFP_2_ uptake was observed. With an intense ^99m^Tc-H-PoFP_2_ uptake with a standardized uptake value maximum (SUVmax) of 3.7, a target-to-background ratio (TBR) of 1.5 was observed throughout the entire lung, corresponding to reticular abnormalities and honeycombing opacities shown on HRCT. Additionally, a solid lesion (2.8 cm × 2.6 cm with SUVmax of 4.2, TBR of 1.7) was identified in the basal parts of the right lung, exhibiting higher uptake than the surrounding fibrosis region. In another case (Figure 4B), markedly increased ^99m^Tc-H-PoFP_2_ uptake (SUVmax 8.2, TBR 3.9) was observed throughout the whole lung, with predominant reticular abnormalities and honeycombing on HRCT. In addition, increased uptake of ^99m^Tc-H-PoFP_2_ around the right knee joint was also observed.

**Figure 5:**
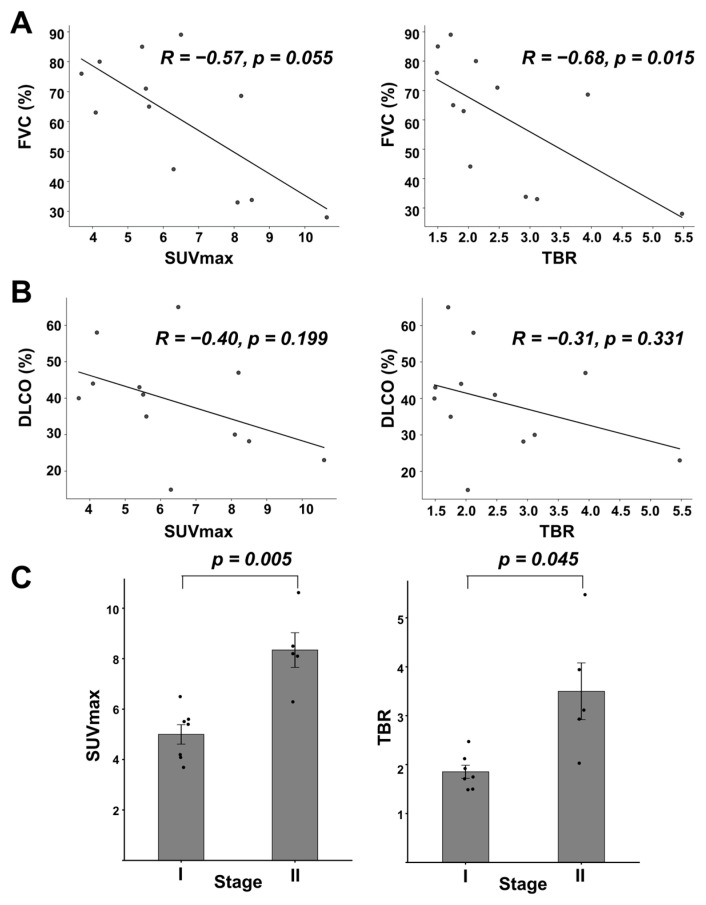
Relationship between ^99m^Tc-H-PoFP_2_ uptake with PFT and GAP index. (**A**) Linear correlation between SUVmax or TBR with FVC (% predicted). For SUVmax, *R* = −0.57, *p* = 0.055; for TBR, *R* = −0.68, *p* = 0.015. (**B**) Linear correlation between SUVmax or TBR with DLCO (% predicted). For SUVmax, *R* = −0.40, *p* = 0.199; for TBR, *R* = −0.31, *p* = 0.331. (**C**) SUVmax or TBR stratified by GAP index. SUVmax was 5.00 ± 1.02 vs. 8.34 ± 1.54 for Stages I and II, respectively (*p* = 0.005). TBRs were 1.85 ± 0.35 vs. 3.50 ± 1.30 for Stages I and II, respectively (*p* = 0.045).

Correlations between SUVmax and TBR with corresponding FVC and DLCO were assessed (Figure 5A,B). SUVmax demonstrated a moderate negative correlation with both FVC and DLCO (*R* = −0.57, *p* = 0.055 for FVC; *R* = −0.40, *p* = 0.199 for DLCO). Similarly, TBR also showed moderated negative correlations with FVC and DLCO as well. Patients were further stratified according to GAP index scores. Stage II patients (n = 5/12; 41.7%) exhibited significantly higher SUVmax and TBR values compared to those in Stage I (n = 7/12; 58.3%) (SUVmax: 5.00 ± 1.02 vs. 8.34 ± 1.54, *p* = 0.005; TBR: 1.85 ± 0.35 vs. 3.50 ± 1.30, *p* = 0.045) (Figure 5C).

## 3. Discussion

Fibrotic interstitial lung disease (fILD) represents a spectrum of chronic lung disorders that share the common feature of progressive fibrosis of the pulmonary parenchyma, leading to significant functional impairment and early mortality [14,15]. The diagnosis of fILD necessitates a multidisciplinary approach, integrating clinical assessments, radiological findings, and sometimes histopathological results. For early diagnosis, HRCT and histology/biopsy exhibit limitations. In contrast, nuclear medicine imaging has been routinely utilized for clinical diagnoses of cancer, cardiovascular, and neuroimaging due to its molecular-level insights and functional assessment [16]. Preclinical studies have demonstrated that activated fibroblasts play a pivotal role in the pathogenesis and progression of the fibrotic process in fILD [17,18,19,20]. Several clinical investigations into FAPI PET imaging in fILD patients have yielded robust and promising results regarding the performance of FAPI PET in disease evaluation, stratification of risk, and response to anti-fibrosis therapy [21,22,23,24]. Investigations into ^99m^Tc-labeled FAPI for SPECT imaging remain limited [25]. There are especially few ^99m^Tc-labeled FAPI tracers for imaging lung fibrosis.

Previously, we reported ^99m^Tc-HFAPI for imaging idiopathic pulmonary fibrosis [7]. However, ^99m^Tc-HFAPI would be decomposed in the body and was mainly excreted through the hepatobiliary system, resulting in a relatively high uptake in the gastrointestinal tract [10]. In this study, we designed a novel ^99m^Tc-labeled FAPI-based radiopharmaceutical with optimized pharmacokinetics, guided by rational structural modifications. Specifically, we adopted the dimerization strategy to enhance the binding affinity to FAP and improve the in vivo stability of the radiopharmaceutical. Meanwhile, we introduced two PEG_4_ linkers to increase the hydrophilicity of the probe, which may shift the primary excretion pathway from hepatobiliary to renal (LogD = −3.56, indicating strong hydrophilicity), reduce gastrointestinal uptake, and lower the background signal. Collectively, these structural modifications endowed ^99m^Tc-H-PoFP_2_ with superior in vivo stability compared to ^99m^Tc-HFAPI. Regarding binding affinity, ^99m^Tc-H-PoFP_2_ showed comparable Kd values to ^99m^Tc-HFAPI (5.35 nM and 4.49 nM) [10]. The dimeric structures theoretically confer higher affinity for FAP than the monomer [26]. The lack of a significant decrease in Kd may be attributed to modifications in the targeting motif between ^99m^Tc-H-PoFP_2_ and ^99m^Tc-HFAPI. Nevertheless, ^99m^Tc-H-PoFP_2_ demonstrated excellent in vivo FAP-targeting specificity in tumor-bearing mice (Figures S3 and S4) and bleomycin-induced lung fibrosis animal models (Figure 2). Critically, the regions with high radioactive signals in SPECT images were consistent with fibrotic foci identified by CT imaging and pathological staining results (HE, Masson, and FAP IHC). Notably, a discrepancy between SPECT and CT signals was observed. Mouse 2 showed stronger CT intensity but lower SPECT signal than Mouse 1 (Figure 2A). This inconsistency may arise from the distinct biological information captured by the imaging modality between CT and SPECT. CT reflects anatomical structural changes associated with fibrosis severity, while ^99m^Tc-H-PoFP_2_ SPECT imaging specially indicates FAP expression. FAP is upregulated in activated fibroblasts during the active phase of fibrosis and its expression decreases as fibrosis progresses to a chronic stage whilst structural fibrosis persists in the bleomycin-induced pulmonary fibrosis mouse model [20,27]. The SPECT/CT imaging was performed on day 14 post-intratracheal injection, and the mouse model should be at the fibrotic stage with high FAP expression. However, the individual mice may exhibit inherent variability during the establishment of the bleomycin-induced fibrosis model [27]. M1 likely remained in the active fibrotic phase with high FAP expression and a strong SPECT signal, while M2 may have transitioned to an early chronic phase with reduced FAP activity, a weak SPECT signal, and ongoing structural fibrosis, which are strong CT signals. Further studies should be conducted to determine the relationship between ^99m^Tc-H-PoFP_2_ SPECT imaging and CT imaging at different stages of pulmonary fibrosis. Furthermore, in another study regarding ^99m^Tc-HFAPI SEPCT/CT imaging for pulmonary fibrosis, the inconsistency was also observed with lower ^99m^Tc-HFAPI uptake in areas of severe lung fibrosis defined by HRCT [7]. This may be attributed to the decreased blood flow in end-stage fibrosis, which affects the ^99m^Tc-FAPI uptake. Finally, several technical factors may also affect ^99m^Tc-H-PoFP_2_ uptake. Given that pulmonary fibrosis lesions exhibit a diffuse rather than focal distribution, and that SPECT has lower spatial resolution, the uptake in diffuse lesions may be filtered out during SPECT data reconstruction, ultimately compromising FAPI uptake and resulting in the inconsistency between CT and SPECT signals.

Based on the excellent performance in preclinical studies, we validated ^99m^Tc-H-PoFP_2_ in 12 fILD patients. No adverse effects were reported during the tracer administration or examination. The average total-body effective dose (0.00724 mSv/MBq) was comparable to other known single photon tracers and lower than that of ^68^Ga or ^18^F-labled FAPI (0.0164 mSv/MBq for ^68^Ga-FAPI-04; 0.00780 mSv/MBq for ^68^Ga-FAPI-46; 0.0124 mSv/MBq for ^18^F-NOTA-FAPI-04) [28,29]. We found accumulated uptake of ^99m^Tc-H-PoFP_2_ in fibrotic lung regions. The markedly increased ^99m^Tc-H-PoFP_2_ regions corresponded well with HRCT patterns of pulmonary fibrosis. Interestingly, ^99m^Tc-H-PoFP_2_ exhibited high uptake around the right knee joint in the patient (Figure 4B) who had experienced right knee pain for several months, potentially indicating arthritis. The high uptake of ^99m^Tc-H-PoFP_2_ suggests the presence of active fibrosis in the joint. Several studies have reported that FAPI PET may assist in diagnosing and monitoring disease activity in rheumatoid arthritis and psoriatic arthritis [30,31]. Interestingly, despite observing extensive honeycombing on HRCT, only mild ^99m^Tc-H-PoFP_2_ uptake was noted in one case (Figure 4A). Experimental studies have found that FAP expression is induced during the early phase of lung fibroblast activation [24]. Pathologic sections of explanted lungs of fILD patients confirmed lower FAP expression in the late-stage fibrotic regions, like honeycombing, where matrix deposition and tissue remodeling predominate rather than being overtaken by ongoing active fibrosis [32]. In addition, the lower resolution of SPECT could reduce its sensitivity as smaller foci of fibroblastic activity could be obscured by larger inactive areas, resulting in reduced or undetectable uptake signals [33]. Thus, the uptake of ^99m^Tc-H-PoFP_2_ might be lower. Additionally, a solid lesion with high ^99m^Tc-H-PoFP_2_ uptake was identified in this patient. Given that lung cancer is a frequent complication of fILD, this lesion should be further clarified. However, due to the deterioration of lung function in this patient, the lesion had not been managed. Different time–activity curves have been observed in the dynamic FAPI PET imaging for fILD and lung cancer lesions [34]. Whether ^99m^Tc-H-PoFP_2_ uptake differs in the fILD and lung cancer lesion could be further investigated. Importantly, ^99m^Tc-H-PoFP_2_ uptake showed moderate negative correlations with PFT parameters, FVC, and DLCO, which were consistent with other FAPI PET studies. Moreover, our study revealed that patients at a higher GAP index stage exhibited significantly higher SUVmax and TBR values compared to those at a lower stage. Studies have demonstrated that the GAP index stratifies patients into different stages (I, II, and III) based on their risk of mortality, with higher scores indicating worse prognosis and predicting mortality in ILD patients [35,36]. Our findings suggest that the ^99m^Tc-H-PoFP_2_ SPECT imaging is not only useful for diagnosing the presence of fILD but also may reflect the severity of lung fibrosis and predict disease progression.

Despite these promising results, there are several limitations of our study. Firstly, we only investigated ^99m^Tc-H-PoFP_2_ SPECT imaging in pulmonary fibrosis models at a single time point, but did not explore whether ^99m^Tc-H-PoFP_2_ SPECT imaging could monitor the progression of pulmonary fibrosis and response to anti-fibrotic treatment. We also did not conduct a biodistribution study in the pulmonary fibrosis mouse model, and thus we lack quantitative distribution data of ^99m^Tc-H-PoFP_2_ in this model. In a subsequent experimental design, we should optimize the study protocol to ensure the comprehensiveness and rigor of the research. Secondly, while we preliminarily verified the feasibility of ^99m^Tc-H-PoFP_2_ SPECT imaging in fILD, our present study is a single-time-point analysis. Further well-designed studies with a larger cohort are warranted to see whether ^99m^Tc-H-PoFP_2_ SPECT imaging could monitor the response to anti-fibrotic therapy. Additionally, although the preclinical data validated the correlation between immunohistochemical expression of FAP and ^99m^Tc-H-PoFP_2_ uptake, the patient study could not confirm this result due to the lack of patients’ tissue biopsy.

## 4. Materials and Methods

### 4.1. Reagents and Materials

All the reagents and solvents were purchased commercially and were of analytical grade. Na^99m^TcO_4_ was purchased from Beijing Atom High-Tech Co., Ltd. (Beijing, China). The radio high-performance liquid chromatography (radio-HPLC) was conducted with an Agilent 1260 HPLC system (Santa Clara, CA, USA). A semi-preparative column (YMC-Pack ODS-AC-18, 250 × 10 mm, 5 μm) and a C18 column (YMC-Pack ODS-A, 250 × 4.6 mml, 5 μm) (YMC Co., Ltd., Kyoto, Japan) were used for chemical synthesis and radiochemical analysis, respectively. FAP was purchased from Sino Biological, Inc. (Beijing, China).

### 4.2. Chemical Synthesis and ^99m^Tc Radiolabeling

The detailed synthesis route and chemical characterization of HYNIC-(PEG_4_-oncoFAPi)_2_ (denoted as H-PoFP_2_) are described in the Supporting Information. The ^99m^Tc-H-PoFP_2_ radiolabeling procedure was performed as follows. In total, 1 mL of Na^99m^TcO_4_ solution was added to a lyophilized kit containing tricine (6.5 mg), trisodium triphenylphosphine-3,3′,3″-trisulfonate (TPPTS) (5 mg), succinic acid (59 mg), sodium hydroxide (20 mg), and H-PoFP_2_ (25 μg). Next, the kit was heated to 100 °C for 20~25 min. After cooling to room temperature, ^99m^Tc-H-PoFP_2_ was analyzed with radio-HPLC. Phase A was phosphate-buffered saline (PBS), and phase B was ACN with 0.05% TFA. The flow rate was 1 mL/min. The gradient mobile phase started from 15% phase B at 0~5 min and progressed to 85% phase B at 20 min and 15% phase B at 25 min. The product was then formulated in PBS and passed through a 0.22 μm Millipore filter (Burlington, MA, USA) into a sterile vial for further use.

### 4.3. Determination of the Partition Coefficient (LogD)

An aliquot of ^99m^Tc-H-PoFP_2_ (74 kBq) was added to a centrifuge tube with 5.0 mL PBS (pH 7.4) and 5.0 mL n-octanol and vortexed for 10 min. After centrifugation (20,000 rpm, 10 min), three samples (500 μL × 3) were taken from each phase and measured in a γ-counter. The octanol/PBS partition coefficient (LogP) was determined as the logarithm of the octanol/PBS ratio. The determination of LogD was carried out twice with triplicate samples. The values of logD are given as mean values ± standard deviation.

### 4.4. In Vivo Stability Determination

For the in vivo stability studies, normal BALB/c mice were injected with ^99m^Tc-H-PoFP_2_ (18 MBq) through the tail vein. Murine urine samples at 30, 120, and 240 min post-injection (p.i.) were collected and mixed with acetonitrile, and then passed through a 0.22 μm Millipore filter. The percentage of intact ^99m^Tc-H-PoFP_2_ was then analyzed by radio-HPLC.

### 4.5. In Vitro Binding Specificity and Affinity Determination

For the protein binding assay, 100 μL of human FAP (2 μg/mL) was coated onto a detachable 96-well plate at 4 °C for 24 h. The coating solution was then removed, the plates were washed three times with cold PBS, and then blocked with 4% BSA for 30 min. For binding specificity determination, ^99m^Tc-H-PoFP_2_ was diluted with PBS and added to the 96-well plate, and incubated at 37 °C for 1 h with or without excess unlabeled precursor H-PoFP_2_ (binding group and block group). After incubation, the plates were washed five times with ice-cold PBS solution containing 1% BSA. These wells were then put into corresponding radioimmunoassay tubes, and the cpm of each well was measured. For binding affinity determination, ^99m^Tc-H-PoFP_2_ was diluted with PBS to prepare solutions of increasing concentration (0−200 nmol/L), which were added to the 96-well plate and incubated at 37 °C for 1 h. After incubation, the plates were washed five times with ice-cold PBS solution containing 1% BSA. The wells were then put into corresponding radioimmunoassay tubes, the cpm of each well was measured, and nonlinear fitting calculations were carried out using GraphPad 9.0 software to obtain the apparent dissociation constant (Kd) of ^99m^Tc-H-PoFP_2_ to human FAP. Four parallel samples were set at each test point, and the experiment was repeated twice.

### 4.6. Establishment of Bleomycin-Induced Lung Fibrosis Model

All animal experiments were performed in accordance with the guidelines of the Institutional Animal Care and Use Committee (IACUC) of Peking Union Medical College Hospital (approval number XHDW-2024-97). C57BL6 mice (female, 6 weeks of age) were purchased from the Department of Animal Experiment, Peking University Health Science Center, and were housed under a 12 h light/12 h dark cycle, with free access to food and water. To establish bleomycin-induced lung fibrotic mouse models, C57/BL6 mice received a single intratracheal injection of bleomycin (2.5~3.5 U/kg, Santa Cruz Biotechnology, Dallas, TX, USA) or saline under anesthesia (3% isoflurane). These mice were imaged at day 14 after intratracheal injection (n = 3 for each group).

### 4.7. SPECT/CT Imaging in Bleomycin-Induced Lung Fibrosis Model

^99m^Tc-H-PoFP_2_ (37 MBq) was injected into the bleomycin-induced lung fibrotic mice and normal mice via the tail vein. At 1 h post-injection, the mice were anesthetized via inhalation of 2% isoflurane and imaged using SPECT/CT. SPECT imaging parameters were as listed: 140 keV photopeak and a 20% window width, with a 30 s frame duration. The parameters of helical CT scans were 50 kVp, 0.67 mA, a 210 rotation, and a 300 ms exposure time. SPECT and CT images were fused using the NanoScan SPECT/CT system by Mediso Ltd. (Budapest, Hungary). Representative CT images and SPECT/CT fused images were shown.

### 4.8. HE and Immunohistochemical Analysis of Fibrotic and Normal Lung Tissues

HE and Masson staining of bleomycin-induced fibrotic and normal lung tissues were performed following standard procedures. For FAP staining, lung tissues were incubated with a rabbit anti-FAP antibody (1:200; ab28244; Abcam, Waltham, MA, USA) overnight at 4 °C. Slide sections were then incubated with an HRP-conjugated goat anti-rabbit IgG antibody (1:200 dilution, GB23303, Servicebio, Wuhan, China) for 1 h and visualized following incubation with diaminobenzidine substrate.

### 4.9. Patients Enrolling and Characteristics

The clinical study was granted approval by the Institutional Review Board of Peking Union Medical College Hospital, Chinese Academy of Medical Sciences, and Peking Union Medical College (Ethics committee approval No. I-22YJ129). Prior to participation, all patients provided written informed consent. The calculation of radiation dosimetry, evaluation of biodistribution, and feasibility of ^99m^Tc-H-PoFP_2_ imaging were conducted in 12 patients diagnosed with fILD. The fILD diagnoses were established by multidisciplinary team discussions based on clinical manifestations and radiologic patterns on HRCT. PFTs were executed in accordance with standardized protocols [37]. Key parameters, including forced vital capacity (FVC) (% predicted) and diffusing capacity of the lungs for carbon monoxide (DLCO) (% predicted), were documented. Additionally, the gender–age–physiology (GAP) index score, which has been used to evaluate the mortality risk in ILD patients, was calculated as well [35]. In brief, the score is derived from four variables, including gender, age, FVC, and DLCO. The cumulative score categorizes patients into Stage I (0–3 points), Stage II (4–5 points), or Stage III (6–8 points) [38].

### 4.10. Whole-Body Scintigraphy and SPECT/CT Imaging Protocol

Sequential whole-body scintigraphy was performed at 5 min, 20 min, 40 min, 1 h, 2 h, 3 h, 4 h, and 6 h post-injection of ^99m^Tc-H-PoFP_2_ (723.9 ± 52.4 MBq) on a dual-head Insight NM/CT Pro SPECT/CT scanner (Novel Medical, Beijing, China). Chest SPECT/CT was conducted approximately 200 ± 20 min post-injection. The SPECT data were normalized and corrected for attenuation, decay, and scatter utilizing an iterative reconstruction algorithm provided by the manufacturer-supplied technique. Patients were asked to report any abnormalities during the examination.

### 4.11. Distribution and Dosimetry Calculation

The overall biodistribution of ^99m^Tc-H-PoFP_2_ was assessed visually. Biodistribution and dosimetry calculation analysis were conducted in six representative patients using HERMES Hybrid Viewer 4.0 Dosimetry (HERMES Medical Solutions) according to OLINDA/EXM (version 2.0) methodology as previously described [39]. In brief, regions of interest (ROIs) were manually delineated over the target organs, including the lungs, heart, liver, kidneys, pancreas, spleen, thyroid, salivary glands, urinary bladder, and residual body. The geometric mean counts were derived from background-corrected anterior and posterior counts. The number of disintegrations and the time–activity curve were generated for each target organ. Absorbed dose of target organs and effective dose were calculated for dosimetry estimation.

### 4.12. Image Analysis and Quantification

^99m^Tc-H-PoFP_2_ SPECT images were visually compared with HRCT findings regarding the sites and extent of tracer uptake. For quantitative or semi-quantitative analysis, a cylindrical radioactive source with well-determined ^99m^Tc activity was used to determine the conversion factors in the volumes of interest to standardized uptake values (SUVs). SUVs for ROIs were computed based on the corresponding patient weight, injected activity, and ^99m^Tc camera calibration factor with HERMES HybridRecon standardized uptake value (SUV) SPECT. SUVmax was recorded based on the highest voxel value within the fibrotic region for each patient. The target-to-background ratio (TBR) was then calculated by normalizing the SUVmax relative to the blood pool as the reference background.

### 4.13. Statistical Analysis

All statistical analysis was executed using GraphPad Prism software, version 9.0.0. (GraphPad Software Inc., San Diego, CA, USA). Data were presented as mean ± standard deviation (SD). Differences between group means were compared using Student’s *t*-test. Correlation analyses were performed using the Spearman correlation test. A *p*-value < 0.05 was considered statistically significant.

## 5. Conclusions

In conclusion, our findings underscore the promising potential of ^99m^Tc-H-PoFP_2_ SPECT imaging in pulmonary fibrosis, which offers a viable noninvasive assessment method for the diagnosis, staging, and prognosis of fILD. Further research is needed to validate these findings and explore the broader clinical applications of ^99m^Tc-H-PoFP_2_ imaging in the management of fILD.

## Figures and Tables

**Table 1:** Baseline demographic data of patients enrolled in the study.

Baseline Demographic Data	N = 12
Age, mean (range; SD)	48.8 (34–65; 11.7)
Gender	
Male/female	3/9
Anti-fibrosis therapy	7
Pulmonary function tests, mean (range; SD)	
FVC (%)	61.4 (28.0–89.0; 21.4)
DLCO (%)	39.1 (14.9–65; 14.1)
SUVmax, mean (range; SD)	6.4 (3.7–10.6; 2.1)
TBR, mean (range; SD)	2.5 (1.5–5.5; 1.2)

Definition of abbreviations: N, number; FVC, forced vital capacity; DLCO, diffusion capacity for carbon monoxide; SD, standard deviation; SUV, standardized uptake value; TBR, target to background ratio. All data were presented as mean ± standard deviation (SD).

**Table 2:** Organ absorbed and effective doses (mSv/MBq) of ^99m^Tc-H-PoFP_2_. (ICRP-103).

Target Organs	Mean	SD
Adrenals	6.74 × 10^−5^	1.57 × 10^−5^
Brain	2.58 × 10^−5^	9.78 × 10^−6^
Breasts	6.51 × 10^−4^	1.96 × 10^−4^
Esophagus	2.21 × 10^−4^	6.34 × 10^−5^
Gallbladder Wall	6.29 × 10^−5^	2.16 × 10^−5^
Left Colon	3.80 × 10^−4^	1.30 × 10^−4^
Small Intestine	1.08 × 10^−4^	8.08 × 10^−5^
Stomach Wall	7.95 × 10^−4^	2.59 × 10^−4^
Right Colon	3.53 × 10^−4^	1.31 × 10^−4^
Rectum	2.24 × 10^−4^	1.01 × 10^−4^
Heart Wall	5.85 × 10^−5^	1.89 × 10^−5^
Kidneys	9.75 × 10^−5^	8.37 × 10^−5^
Liver	1.67 × 10^−4^	5.88 × 10^−5^
Lungs	8.37 × 10^−4^	6.51 × 10^−4^
Ovaries	3.30 × 10^−4^	1.85 × 10^−4^
Pancreas	5.93 × 10^−5^	2.65 × 10^−5^
Prostate	5.02 × 10^−5^	3.66 × 10^−5^
Salivary Glands	5.06 × 10^−5^	1.95 × 10^−5^
Red Marrow	6.86 × 10^−4^	2.31 × 10^−4^
Osteogenic Cells	1.01 × 10^−4^	3.53 × 10^−5^
Spleen	6.05 × 10^−5^	1.95 × 10^−5^
Testes	2.11 × 10^−4^	1.05 × 10^−4^
Thymus	6.41 × 10^−5^	2.22 × 10^−5^
Thyroid	7.03 × 10^−4^	1.36 × 10^−3^
Urinary Bladder Wall	1.26 × 10^−3^	1.30 × 10^−3^
Uterus	1.77 × 10^−4^	1.86 × 10^−4^
Effective Dose	7.24 × 10^−3^	1.60 × 10^−3^

## Data Availability

The original contributions presented in this study are included in the article/Supplementary Material. Further inquiries can be directed to the corresponding authors.

## Supplementary Materials

The following supporting information can be downloaded at https://www.mdpi.com/article/10.3390/ph18121779/s1. Figure S1: Synthesis route of HYNIC-(PEG_4_-oncoFAP)_2_ (denoted as H-PoFP_2_); Figure S2: Representative HPLC chromatogram images and TOF MS results of HYNIC-(PEG_4_-oncoFAP)_2_ and related compounds; Figure S3: SPECT/CT imaging of ^99m^Tc-H-PoFP_2_ in U87MG tumor-bearing mice; Figure S4: Ex vivo biodistribution of ^99m^Tc-H-PoFP_2_ in U87MG tumor-bearing mice; Figure S5. Blood clearance rate curve of 99mTc-H-PoFP2 in ICR mice; Figure S6. Whole-body planar SPECT/CT images of bleomycin-induced pulmonary fibrosis mice (M1, M2, and M4) and normal mice (M3, M5, and M6); Table S1: Detailed biodistribution data ^99m^Tc-H-PoFP_2_ in U87MG tumor-bearing mice.

## References

[1] Maher T.M. (2024). Interstitial Lung Disease: A Review. JAMA.

[2] Wijsenbeek M., Cottin V. (2020). Spectrum of Fibrotic Lung Diseases. N. Engl. J. Med..

[3] Muri J., Durcová B., Slivka R., Vrbenská A., Makovická M., Makovický P., Škarda J., Delongová P., Kamarád V., Vecanová J. (2024). Idiopathic Pulmonary Fibrosis: Review of Current Knowledge. Physiol. Res..

[4] Allison M.B., Catana C., Zhou I.Y., Caravan P., Montesi S.B. (2025). Molecular Imaging of Pulmonary Fibrosis. J. Nucl. Med..

[5] Kelly T., Huang Y., Simms A.E., Mazur A. (2012). Fibroblast Activation Protein-α: A Key Modulator of the Microenvironment in Multiple Pathologies. Int. Rev. Cell Mol. Biol..

[6] Bundgaard-Nielsen M., Johnsen R.H., Mortensen J., Shaker S.B., Nielsen C.T.H. (2025). Radio-Labelled Fibroblast Activation Protein Inhibitors in Interstitial Lung Diseases—A Systematic Review. Autoimmun. Rev..

[7] Liu Y., Zhang Q., Zhang Y., Wang J., Wu Y., Yang G., Shi J., Wang F., Xu Z., Jing H. (2023). 99mTc-Labeled FAPI SPECT Imaging in Idiopathic Pulmonary Fibrosis: Preliminary Results. Pharmaceuticals.

[8] Yang G., Gao H., Luo C., Zhao X., Luo Q., Shi J., Wang F. (2022). Palmitic Acid-Conjugated Radiopharmaceutical for Integrin Avβ3-Targeted Radionuclide Therapy. Pharmaceutics.

[9] Zhao L., Niu B., Fang J., Pang Y., Li S., Xie C., Sun L., Zhang X., Guo Z., Lin Q. (2022). Synthesis, Preclinical Evaluation, and a Pilot Clinical PET Imaging Study of 68Ga-Labeled FAPI Dimer. J. Nucl. Med..

[10] Ma M., Yang G., Zhao M., Liu Y., Ge X., Jia B., Gao S. (2024). Synthesis and Preliminary Study of 99mTc-Labeled HYNIC-FAPi for Imaging of Fibroblast Activation Proteins in Tumors. Mol. Pharm..

[11] Ruan Q., Diao L., Li Z., Ding D., Han P., Jiang Y., Yin G., Feng J., Wang Q., Jiang J. (2025). Design and Preclinical Evaluation of 99mTc-Labeled Dimer FAPI-46 Derivatives as Potential Tumor Radiotracers. Eur. J. Med. Chem..

[12] Meng L., Fang J., Zhang J., Li H., Xia D., Zhuang R., Chen H., Huang J., Li Y., Zhang X. (2024). Rational Design and Comparison of Novel 99mTc-Labeled FAPI Dimers for Visualization of Multiple Tumor Types. J. Med. Chem..

[13] Stokke C., Gnesin S., Tran-Gia J., Cicone F., Holm S., Cremonesi M., Blakkisrud J., Wendler T., Gillings N., Herrmann K. (2024). EANM Guidance Document: Dosimetry for First-in-Human Studies and Early Phase Clinical Trials. Eur. J. Nucl. Med. Mol. Imaging.

[14] Goobie G.C., Marinescu D.-C., Adegunsoye A., Bourbeau J., Carlsten C., Clifford R.L., Doiron D., Duan Q., Gibson K.F., Grant-Orser A. (2025). Accelerated Epigenetic Aging Worsens Survival and Mediates Environmental Stressors in Fibrotic Interstitial Lung Disease. Eur. Respir. J..

[15] Wijsenbeek M., Suzuki A., Maher T.M. (2022). Interstitial Lung Diseases. Lancet.

[16] Sharma P., Singh S.S., Gayana S. (2021). Fibroblast Activation Protein Inhibitor PET/CT: A Promising Molecular Imaging Tool. Clin. Nucl. Med..

[17] Walsh S.L.F., Wells A.U., Sverzellati N., Devaraj A., von der Thüsen J., Yousem S.A., Colby T.V., Nicholson A.G., Hansell D.M. (2015). Relationship between Fibroblastic Foci Profusion and High Resolution CT Morphology in Fibrotic Lung Disease. BMC Med..

[18] Harada T., Watanabe K., Nabeshima K., Hamasaki M., Iwasaki H. (2013). Prognostic Significance of Fibroblastic Foci in Usual Interstitial Pneumonia and Non-Specific Interstitial Pneumonia. Respirology.

[19] Egger C., Cannet C., Gérard C., Suply T., Ksiazek I., Jarman E., Beckmann N. (2017). Effects of the Fibroblast Activation Protein Inhibitor, PT100, in a Murine Model of Pulmonary Fibrosis. Eur. J. Pharmacol..

[20] Rosenkrans Z.T., Massey C.F., Bernau K., Ferreira C.A., Jeffery J.J., Schulte J.J., Moore M., Valla F., Batterton J.M., Drake C.R. (2022). 68Ga-FAPI-46 PET for Non-Invasive Detection of Pulmonary Fibrosis Disease Activity. Eur. J. Nucl. Med. Mol. Imaging.

[21] Albu M.T., Matei A.-E., Distler J.H.W., Giesel F.L., Mori Y. (2024). Fibroblast Activation Protein Inhibitor PET/CT as an Emerging Diagnostic Modality in Interstitial Lung Disease and Other Fibrotic Conditions. Rheumatol. Immunol. Res..

[22] Bergmann C., Distler J.H.W., Treutlein C., Tascilar K., Müller A.-T., Atzinger A., Matei A.-E., Knitza J., Györfi A.-H., Lück A. (2021). 68Ga-FAPI-04 PET-CT for Molecular Assessment of Fibroblast Activation and Risk Evaluation in Systemic Sclerosis-Associated Interstitial Lung Disease: A Single-Centre, Pilot Study. Lancet Rheumatol..

[23] Kastrati K., Nakuz T.S., Kulterer O.C., Geßl I., Simader E., Mrak D., Bonelli M., Kiener H.P., Prayer F., Prosch H. (2024). FAPi PET/CT for Assessment and Visualisation of Active Myositis-Related Interstitial Lung Disease: A Prospective Observational Pilot Study. EClinicalMedicine.

[24] Yang P., Luo Q., Wang X., Fang Q., Fu Z., Li J., Lai Y., Chen X., Xu X., Peng X. (2023). Comprehensive Analysis of Fibroblast Activation Protein Expression in Interstitial Lung Diseases. Am. J. Respir. Crit. Care Med..

[25] Boschi A., Urso L., Uccelli L., Martini P., Filippi L. (2024). 99mTc-Labeled FAPI Compounds for Cancer and Inflammation: From Radiochemistry to the First Clinical Applications. EJNMMI Radiopharm. Chem..

[26] Younis M.H., Lan X., Cai W. (2022). PET with a 68Ga-Labeled FAPI Dimer: Moving towards Theranostics. J. Nucl. Med..

[27] Moeller A., Ask K., Warburton D., Gauldie J., Kolb M. (2008). The Bleomycin Animal Model: A Useful Tool to Investigate Treatment Options for Idiopathic Pulmonary Fibrosis?. Int. J. Biochem. Cell Biol..

[28] Wang S., Zhou X., Xu X., Ding J., Liu S., Hou X., Li N., Zhu H., Yang Z. (2021). Clinical Translational Evaluation of Al18F-NOTA-FAPI for Fibroblast Activation Protein-Targeted Tumour Imaging. Eur. J. Nucl. Med. Mol. Imaging.

[29] Bentestuen M., Nalliah S., Stolberg M.M.K., Zacho H.D. (2024). How to Perform FAPI PET? An Expedited Systematic Review Providing a Recommendation for FAPI PET Imaging with Different FAPI Tracers. Semin. Nucl. Med..

[30] Zhang Z., Yang L., Jiang L., Tian R., Li Q. (2025). 68Ga-FAPI-04 PET/CT Imaging in a Patient With Psoriatic Arthritis. Clin. Nucl. Med..

[31] Luo Y., Pan Q., Zhou Z., Li M., Wei Y., Jiang X., Yang H., Li F. (2023). 68Ga-FAPI PET/CT for Rheumatoid Arthritis: A Prospective Study. Radiology.

[32] Hotta M., Kim G.H.J., Rerkpichaisuth V., Teng P.Y., Armstrong W.R., Carlucci G., Dahlbom M., Abtin F., Lari S.M., Fishbein G.A. (2024). Correlation of FAPI PET Uptake with Immunohistochemistry in Explanted Lungs from Patients with Advanced Interstitial Lung Disease. J. Nucl. Med..

[33] Alqahtani F.F. (2023). SPECT/CT and PET/CT, Related Radiopharmaceuticals, and Areas of Application and Comparison. Saudi Pharm. J..

[34] Röhrich M., Leitz D., Glatting F.M., Wefers A.K., Weinheimer O., Flechsig P., Kahn N., Mall M.A., Giesel F.L., Kratochwil C. (2022). Fibroblast Activation Protein–Specific PET/CT Imaging in Fibrotic Interstitial Lung Diseases and Lung Cancer: A Translational Exploratory Study. J. Nucl. Med..

[35] Ryerson C.J., Vittinghoff E., Ley B., Lee J.S., Mooney J.J., Jones K.D., Elicker B.M., Wolters P.J., Koth L.L., King T.E. (2014). Predicting Survival across Chronic Interstitial Lung Disease: The ILD-GAP Model. Chest.

[36] Ley B., Ryerson C.J., Vittinghoff E., Ryu J.H., Tomassetti S., Lee J.S., Poletti V., Buccioli M., Elicker B.M., Jones K.D. (2012). A Multidimensional Index and Staging System for Idiopathic Pulmonary Fibrosis. Ann. Intern. Med..

[37] Graham B.L., Brusasco V., Burgos F., Cooper B.G., Jensen R., Kendrick A., MacIntyre N.R., Thompson B.R., Wanger J. (2017). 2017 ERS/ATS Standards for Single-Breath Carbon Monoxide Uptake in the Lung. Eur. Respir. J..

[38] Fujii H., Hara Y., Saigusa Y., Tagami Y., Murohashi K., Nagasawa R., Aoki A., Izawa A., Seki K., Watanabe K. (2023). ILD-GAP Combined with the Charlson Comorbidity Index Score (ILD-GAPC) as a Prognostic Prediction Model in Patients with Interstitial Lung Disease. Can. Respir. J..

[39] Cheng L., Liu F., Gao L., Sun L., Hou Y., Liu Y. An Integrated Framework of Projection and Attenuation Correction for Quantitative SPECT/CT Reconstruction. Proceedings of the 2021 IEEE Nuclear Science Symposium and Medical Imaging Conference (NSS/MIC).

