# Supplementary material for: A Novel FAPI-Based Radiopharmaceutical for SPECT Imaging of Fibrotic Interstitial Lung Disease
Source: Pharmaceuticals (Basel). 2025 Nov 23;18(12):1779. doi: 10.3390/ph18121779 (PMC12735806; doi:10.3390/ph18121779)
Supplement: Supplementary file 1 [file pharmaceuticals-18-01779-s001.zip › pharmaceuticals-3973569-supplementary.pdf]

Supporting Information for ORIGINAL ARTICLE

A Novel FAPI-Based Radiopharmaceutical for SPECT Imaging of Fibrotic Interstitial Lung Disease

Guangjie Yang [1,†], Jingnan Wang [1,†], Yu Liu [1], Jiyun Shi [2], Xueyang Zhang [3], Yangzhong Zhou [3], Qian Wang [3,*], Fan Wang [2,4] and Li Huo [1,*]

[1] Department of Nuclear Medicine, State Key Laboratory of Complex, Severe, and Rare Diseases, Center for Rare Diseases Research, Peking Union Medical College Hospital, Chinese Academy of Medical Science and Peking Union Medical College, Beijing 100730, China; yangguangjie@pumch.cn (G.Y.); jingnanwang1991@163.com (J.W.); liuyu@pumch.cn (Y.L.)

[2] State Key Laboratory of Biomacromolecules, Institute of Biophysics, Chinese Academy of Sciences, Beijing 100101, China; shijiyun@med.uestc.edu.cn (J.S.); wangfan@bjmu.edu.cn (F.W.)

[3] Department of Rheumatology and Clinical Immunology, State Key Laboratory of Complex, Severe, and Rare Diseases, Peking Union Medical College Hospital, Chinese Academy of Medical Sciences and Peking Union Medical College, Beijing 100730, China; zhxy97@outlook.com (X.Z.); hubertchow09@163.com (Y.Z.)

[4] Medical Isotopes Research Center and Department of Radiation Medicine, School of Basic Medical Sciences, International Cancer Institute, Peking University, Beijing 100191, China

* Correspondence: wangqian_pumch@126.com (Q.W.); huoli@pumch.cn (L.H.)

† These authors contributed equally to this work.

1. Synthesis of HYNIC-[PEG$_4$-OncoFAPI]$_2$ (denoted as H-PoFP$_2$)

The synthesis route of HYNIC-(PEG4-oncoFAP)$_2$ (denoted as H-PoFP$_2$) was shown in **Figure S1**. The separation and purification of the related compounds were carried out using an Agilent HPLC 1260 Infinity system equipped with a YMC-Pack ODS-AC-18 semi-preparative column (250 × 10 mm, 5 µm). Phase A was deionized water (containing 0.05% trifluoroacetic acid, TFA), and phase B was acetonitrile (containing 0.05% TFA). The reaction was monitored and the target product was separated and purified by high-performance liquid chromatography (HPLC) with the following method: at 0 min, the mobile phase consisted of 90% A and 10% B; at 20 min, 40% A and 60% B; at 25 min, 90% A and 10% B, with a flow rate of 3.2 mL/min.

Synthesis of oncoFAP (compound 5): Add 8-Aminoquinoline-4-carboxylic acid (compound 1, 1 eq), (S)-1-(2-Aminoacetyl)-4,4-difluoropyrrolidine-2-carbonitrile hydrochloride (compound 2, 1 eq), and O-(7-Azabenzotriazol-1-yl)-N,N,N',N'-tetramethyl uronium hexafluorophosphate (HATU, 1 eq) into a 25-mL round-bottom flask. Dissolve them in 900 µL N,N-dimethylformamide (DMF) and 4 mL of dichloromethane (DCM). Then, add N,N-diisopropylethylamine (DIEA, 4 eq) dropwise and stir for 1 h. Dilute the crude product with DCM, wash with water, dry it with Na_2SO_4, filter it, and finally remove the solvent using a rotary evaporator to obtain the crude product (S)-8-amino-N-(2-(2-cyano-4,4-difluoropyrrolidin-1-yl)-2-oxoethyl) quinoline-4-carboxamide (compound 3). Add the above-mentioned crude product (compound 3, 1 eq), succinic anhydride (compound 4, 50 eq), and 4-(dimethylamino) pyridine (DAMP, 0.5 eq) into a 25-mL round-bottom flask. Dissolve them in 3 mL of tetrahydrofuran (THF) and react at 60 °C for 6 hours. After removing the solvent from the reaction mixture using a rotary evaporator, dilute it with water, extract it with DCM, dry it with Na_2SO_4, filter it, and dry it to obtain (*S*)-

4-((4-((2-(2-cyano-4,4-difluoropyrrolidin-1-yl)-2-oxoethyl)carbamoyl)quinolin-8-yl)amino)-4-oxobutanoic acid (oncoFAP, compound 5). The product was identified by time-of-flight mass spectrometry (TOF MS, ESI^+).

Synthesis of NH_2-PEG$_4$-oncoFAP (compound 7): Dissolve oncoFAP (compound 5, 1 eq) and HATU (1.5 eq) in 200 μL DMF, add DIEA (2 eq) and react at room temperature for approximately 30 minutes. Then, add *tert*-butyl (14-amino-3,6,9,12-tetraoxatetradecyl)carbamate (Boc-NH-PEG$_4$-NH_2, 1 eq) to the above reaction solution. Add DIEA to adjust the pH to 8.5 - 9.0, and react overnight at room temperature. Collect the eluate with a retention time of 20.4 minutes, and lyophilize it using the vacuum freeze-drying method to obtain the expected product *tert*-butyl (*S*)-(19-((4-((2-(2-cyano-4,4-difluoropyrrolidin-1-yl)-2-oxoethyl)carbamoyl)quinolin-8-yl)amino)-16,19-dioxo-3,6,9,12-tetraoxa-15-azanonadecyl)carbamate (Boc-NH-PEG$_4$-oncoFAP, compound 6). Dissolve the product Boc-NH-PEG$_4$-oncoFAP (compound 6) in 1 mL of trifluoroacetic acid (TFA) and react at room temperature for 10 minutes. After blowing dry the reaction solution with nitrogen, dissolve it in 50% acetonitrile/water, purify the product by HPLC, collect the eluate with a retention time of 14.0 minutes, and lyophilize it using the vacuum freeze-drying method. Then (*S*)-*N*1-(14-amino-3,6,9,12-tetraoxatetradecyl)-*N*4-(4-((2-(2-cyano-4,4-difluoropyrrolidin-1-yl)-2-oxoethyl)carbamoyl)quinolin-8-yl)succinimide (NH_2-Glu-PEG$_4$-oncoFAP, compound 7) was afforded. The obtained product was identified by time-of-flight mass spectrometry (TOF MS, ESI^+).

Synthesis of NH_2-Glu-(PEG$_4$-oncoFAP)$_2$ (compound 9): Weigh NH_2-Glu-PEG$_4$-oncoFAP (compound 7, 1 eq) and bis(2,5-dioxopyrrolidin-1-yl) (*tert*-butoxycarbonyl)-*D*-glutamate (Boc-Glu(OSu)-Osu, 0.5 eq), dissolve them in 200 μL DMF. Add DIEA to adjust the pH to 8.5-9.0, and react overnight at room temperature. Collect the eluate with a retention time of 19.9 minutes, and lyophilize the eluate using the vacuum freeze-drying method to obtain the expected product *tert*-butyl ((*R*)-1,45-bis((4-((2-((*S*)-2-cyano-4,4-difluoropyrrolidin-1-yl)-2-oxoethyl)carbamoyl)quinolin-8-yl)amino)-1,4,21,25,42,45-hexaoxo-7,10,13,16,28,31,34,37-octaoxa-5,20,26,41-tetraazapentatetracontan-24-yl)carbamate (Boc-Glu-(PEG$_4$-oncoFAP)$_2$, compound 8) Dissolve the lyophilized product (compound 8) in 1 mL TFA/DCM and react at room temperature for 10 minutes. After blowing dry the reaction solution with nitrogen, dissolve it in 50% acetonitrile/water (ACN/H_2O), purify the product by HPLC, collect the eluate with a retention time of 17.6 minutes, and lyophilize it using the vacuum freeze-drying method. The obtained product, (*R*)-2-amino-*N*1-(19-((4-((2-((*S*)-2-cyano-4,4-difluoropyrrolidin-1-yl)-2-oxoethyl)carbamoyl)quinolin-8-yl)amino)-16,19-dioxo-2,5,8,11-tetraoxa-15-azanonadecyl)-*N*5-(19-((4-((2-((*S*)-2-cyano-4,4-difluoropyrrolidin-1-yl)-2-oxoethyl)carbamoyl)quinolin-8-yl)amino)-16,19-dioxo-4,7,10,13-tetraoxa-15-azanonadecyl)pentanediamide (NH_2-Glu-(PEG$_4$-oncoFAP)$_2$, compound 9), was identified by time-of-flight mass spectrometry (TOF MS, ESI^+).

Synthesis of HYNIC-(PEG$_4$-oncoFAP)$_2$: Dissolve NH$_2$-Glu-(PEG4-oncoFAP)$_2$ (compound 9, 1 eq) and (*E*)-2-((2-(5-(((2,5-dioxopyrrolidin-1-yl)oxy)carbonyl)pyridin-2-yl)hydrazineylidene)methyl) benzenesulfonic acid (HYNIC-NHS, 1 eq) in 100 μL DMF. Adjust the pH to 8.5–9.0 by adding DIEA, and react overnight at room temperature. Collect the eluate with a retention time of 17.63 minutes and lyophilize the eluate via vacuum freeze-drying. The final product (HYNIC-(PEG$_4$-oncoFAP)$_2$) was analyzed by TOF MS (ESI$^+$).

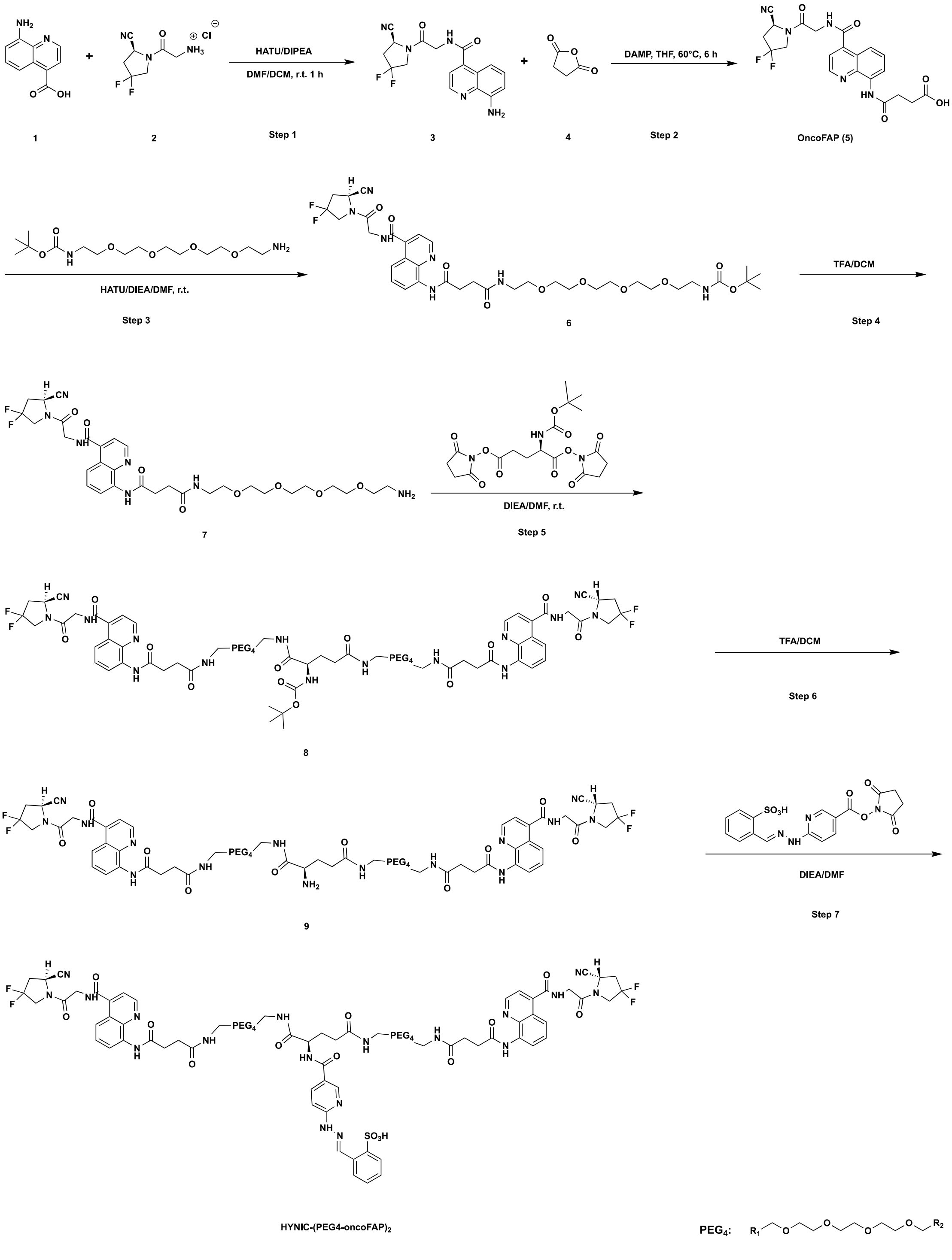

Figure S1. Synthesis route of HYNIC-(PEG4-oncoFAP)$_2$ (denoted as H-PoFP$_2$).

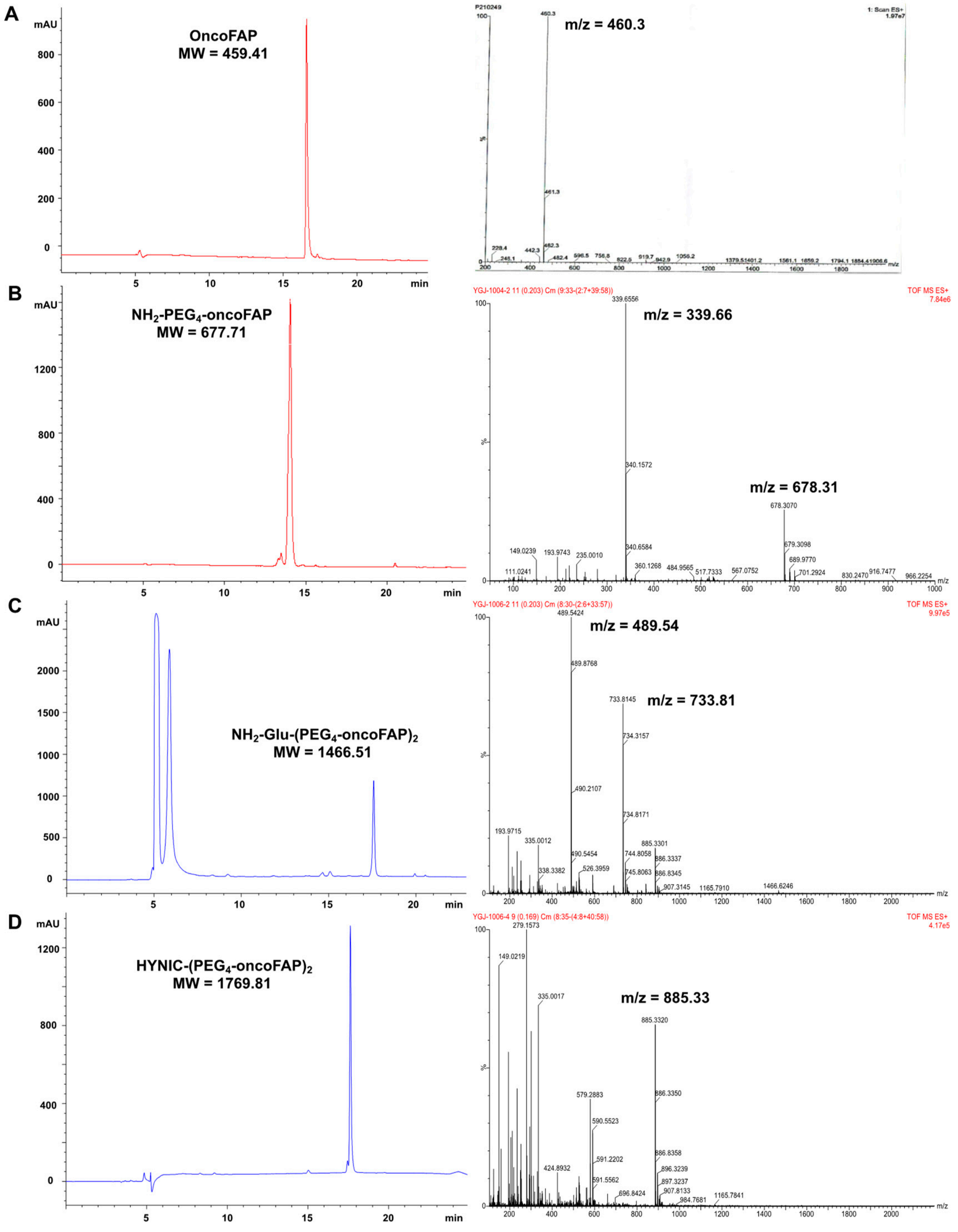

Figure S2. Representative HPLC chromatogram images and TOF MS results of HYNIC-(PEG_4-oncoFAP)$_2$ and related compounds (compound 5, OncoFAP; NH_2-Glu-PEG_4-oncoFAP, compound 7; NH_2-Glu-(PEG_4-oncoFAP)$_2$, compound 9).

The representative HPLC chromatogram images and TOF MS results of HYNIC-(PEG4-oncoFAP)$_2$ and related compounds were shown in **Figure S2**. The synthesis results of oncoFAP (compound 5) are shown in Figure S2A. The product is brown powdery solid. The theoretical molecular weight of oncoFAP

(compound 5) is 459.41, and the result of TOF MS (ESI+) identification is m/z = 460.3 ($[M + H]^+$), confirming it as the expected product.

The synthesis results of NH_2-PEG_4-oncoFAP (compound 7) are shown in Figure S2B. The product is light yellow powdery solid. The theoretical molecular weight of NH_2-PEG_4-oncoFAP (compound 7) is 677.71, and the result of TOF MS (ESI+) identification is m/z = 678.31 ($[M + H]^+$), confirming it as the expected product.

The synthesis results of NH_2-Glu(PEG_4-oncoFAP$)_2$ (compound 9) are shown in Figure S2C. The product is light yellow powdery solid. The theoretical molecular weight of NH_2-Glu(PEG_4-oncoFAP$)_2$ (compound 9) is 1466.51, and the result of TOF MS (ESI+) identification is m/z = 733.81 ($[M + H]^+$), confirming it as the expected product.

The synthesis results of HYNIC-(PEG_4-oncoFAP$)_2$ are shown in Figure S2D. The product is light yellow powdery solid. The theoretical molecular weight of HYNIC-(PEG_4-oncoFAP$)_2$ is 1769.81, and the result of TOF MS (ESI+) identification is m/z = 885.33 ($[M + H]^+$), confirming it as the expected product.

2. SPECT/CT imaging study of ^{99m}Tc-H-PoFP_2 in U87MG tumor bearing mice model

The human glioma cell line U87MG (ATCC® HTB-14™) was purchased from the American Type Culture Collection (Manassas, VA, USA) and cultured in DMEM with 10% fetal bovine serum (FBS) at 37°C in a humidified atmosphere containing 5% CO_2. BALB/c nude mice (Female, 6 weeks of age) were purchased from the Department of Animal Experiment, Peking University Health Science Center and were housed under a 12-h light/12-h dark cycle, with free access to food and water. To establish the U87MG subcutaneous tumor models, U87MG (2.5×10^6) cells were inoculated subcutaneously into the front flanks of BALB/c nude mice. When the tumors reached a size of 200–300 mm^3, the mice were used for biodistribution or SPECT/CT imaging studies. The SPECT/CT imaging study was performed using a small-animal SPECT/CT imaging system (Mediso Inc. Hungary). Each tumor-bearing mouse was injected with ^{99m}Tc-H-PoFP_2 (37 MBq) via the tail vein. The blocking study was performed by co-injecting excess unlabeled FAPI. At 0.5, 1, 2, and 4 h post injection, the mice were anesthetized via inhalation of 2% isoflurane and imaged using SPECT/CT. The pinhole SPECT images (peak 140 keV; 20% width; frame time, 30 s) were acquired, and CT images were subsequently acquired (50 kV; 0.67 mA; rotation, 210°; exposure time, 300 ms). The raw data were reconstructed into a whole-body region. The SPECT and CT images were then fused using Nucline v 2.01 (Mediso Inc. Hungary). The maximum intensity projection (MIP) was obtained for the whole-body imaging using the posterior view. Representative MIP images taken at different times points p.i. were presented in **Figure S3**. ^{99m}Tc-H-PoFP_2 showed remarkable uptake in the tumor and could be blocked significantly by co-injection with unlabeled FAPI, suggesting the uptake in U87MG tumors was target-specific. Besides, ^{99m}Tc-H-PoFP_2

was eliminated mainly through kidney, resulting high uptake in kidney and strong signal in bladder. On the contrast, the uptake of ^{99m}Tc-H-PoFP$_2$ in normal organs was much lower and thus the tumor-background ratio was relatively high.

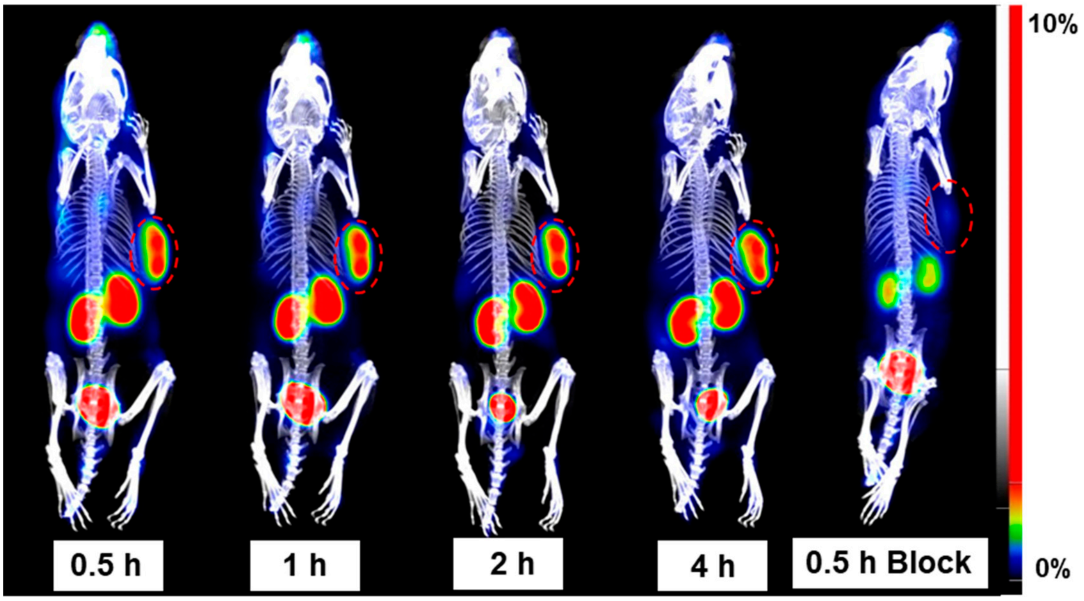

Figure S3. SPECT/CT imaging of ^{99m}Tc-H-PoFP$_2$ in U87MG tumor-bearing mice. U87MG tumors were indicated by red dashed circles.

3. Ex vivo biodistribution study of ^{99m}Tc-H-PoFP$_2$ in U87MG tumor bearing mice model

The biodistribution properties of ^{99m}Tc-H-PoFP$_2$ were determined in U87MG subcutaneous tumor-bearing mice (n = 4 per group). Each tumor-bearing mouse was injected with 370 kBq of ^{99m}Tc-H-PoFP$_2$ via the tail vein and was killed at 0.5 and 4 h after injection. Blood, tumor, and other organs of interest were harvested, weighed, and measured in a γ-counter. Blocking studies were also performed by co-injecting excess unlabeled FAPI. Organ uptake is presented as the percentage of injected dose per gram of tissue (%ID/g). The biodistribution studies were also performed and results were shown in **Figure S4 and Table S1**. In accordance with the SPECT imaging, ^{99m}Tc-H-PoFP$_2$ showed the high uptake and long retention in U87MG tumors at 0.5 h and 4 h p.i. with the value of 26.05 ± 5.39 and 22.51 ± 7.01 %ID/g, respectively. The kidney also showed relatively high uptake, with the value of 10.99 ±2.60 and 13.81 ±0.17 %ID/g at 0.5 h and 4 h p.i.. Meanwhile, we also observed uptake in the knee joints with the value of 6.17 ±1.60 and 4.28 ±0.33 %ID/g at 0.5 h and 4 h p.i.. In the block study at 0.5 h p.i., the uptake in the tumor and knee joints could be blocked significantly (3.94 ± 1.03 %ID/g, P = 0.0022 for tumor; 1.18 ± 0.18 %ID/g, P = 0.0022 for knee joints), indicating the uptake was target-specific.

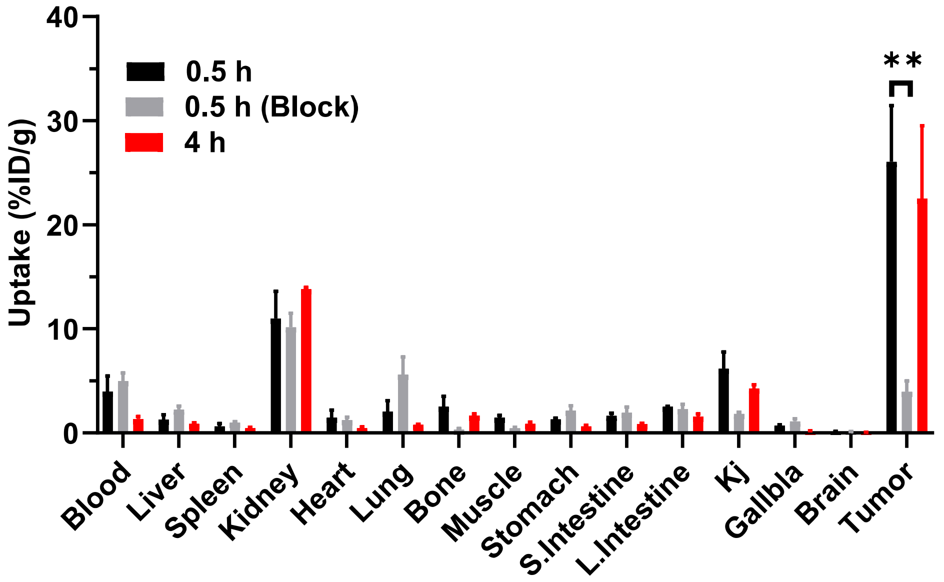

Figure S4. Ex vivo biodistribution of ^{99m}Tc-H-PoFP$_2$ in U87MG tumor-bearing mice. S.intestine, small intestine; L.intestine, large intestine; Kj, knee joint; Gallbla, gall bladder.

Table S1. Detailed biodistribution data of ^{99m}Tc-H-PoFP$_2$ in U87MG tumor-bearing mice.

Group / Organs	0.5 h		0.5 h (Block)		P Value	4 h	
	Mean	SD	Mean	SD		Mean	SD
**Blood**	3.96	1.51	4.98	0.78	0.356278	1.35	0.22
**Liver**	1.28	0.46	2.25	0.34	0.042348	0.89	0.09
**Spleen**	0.64	0.24	0.98	0.12	0.090966	0.47	0.09
**Kidney**	10.99	2.60	10.14	1.36	0.643368	13.81	0.17
**Heart**	1.46	0.74	1.22	0.29	0.629691	0.47	0.10
**Lung**	2.05	1.04	5.62	1.67	0.034717	0.77	0.07
**Bone**	2.51	1.01	0.28	0.13	0.019027	1.69	0.16
**Muscle**	1.45	0.24	0.43	0.13	0.002986	0.89	0.14
**Stomach**	1.31	0.11	2.12	0.50	0.049549	0.63	0.09
**S.Intestine**	1.66	0.23	1.93	0.55	0.472905	0.85	0.11
**L.Intestine**	2.53	0.03	2.26	0.49	0.394002	1.58	0.26

**Kj**	6.17	1.60	1.81	0.18	0.009385	4.28	0.33
**Gallbla**	0.70	0.07	1.13	0.24	0.102342	0.11	0.10
**Brain**	0.13	0.03	0.10	0.04	0.334695	0.04	0.02
**Tumor**	26.05	5.39	3.94	1.03	0.002221	22.51	7.01

4. Blood clearance rate of ^{99m}Tc-H-PoFP$_2$ in normal mice

Female ICR mice (n=5) were injected intravenously with 0.74 MBq of ^{99m}Tc-H-PoFP$_2$. The blood samples obtained from the canthus vein were collected at different time points post injection (p.i.), weighed, and counted using a γ-counter. The results were presented as percentage injected dose per gram (%ID/g) and analyzed by nonlinear regression of two-phase decay by GraphPad Prism 9.0. The blood clearance rate curve of ^{99m}Tc-H-PoFP$_2$ was shown in **Figure S5**. ^{99m}Tc-H-PoFP$_2$ showed a particularly fast blood elimination ($T_{1/2\alpha}$=3.02 min; $T_{1/2\beta}$=166.10 min).

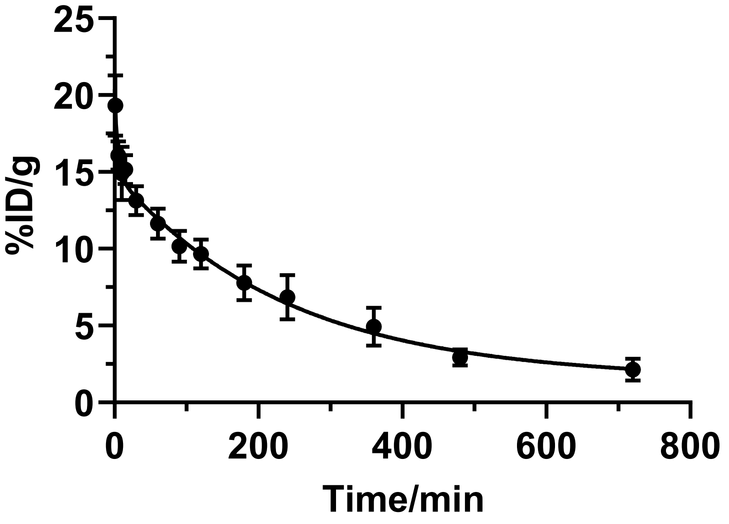

Figure S5. Blood clearance rate curve of ^{99m}Tc-H-PoFP$_2$ in ICR mice.

5. Whole-body planar SPECT/CT images of bleomycin-induced pulmonary fibrosis mice and normal mice.

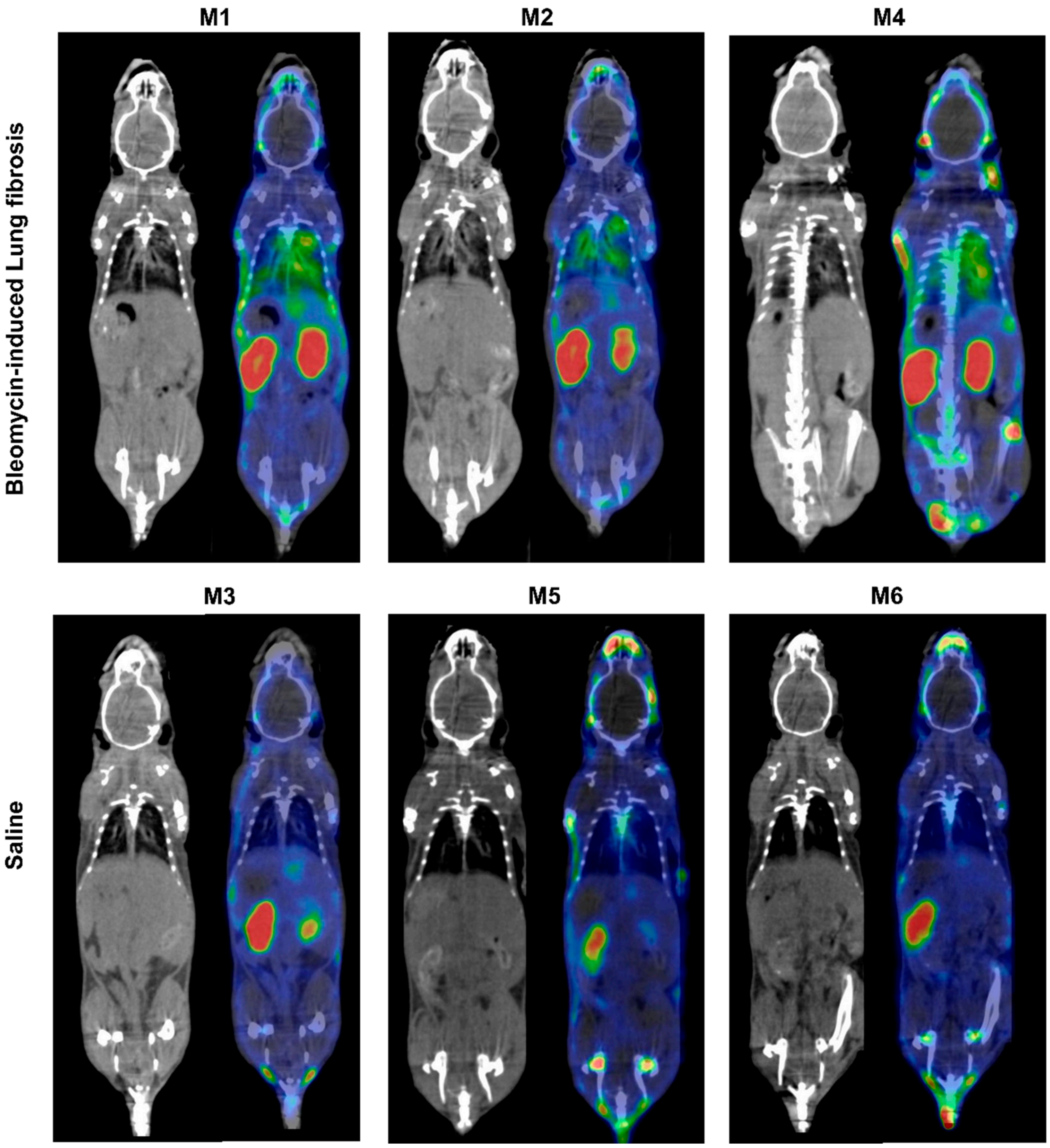

Figure S6. Whole-body planar SPECT/CT images of bleomycin-induced pulmonary fibrosis mice (M1, M2, M4) and normal mice (M3, M5, M6).
